# An Enriched Primary Culture Reveals Morphological Heterogeneity of Rat Testicular Telocytes

**DOI:** 10.3390/cells15171569

**Published:** 2026-08-29

**Authors:** Piotr Pawlicki, Anna Gałuszka, Izabela Siemińska, Adrianna Gałuszka-Bulaga, Ewa Ocłoń

**Affiliations:** 1 Department of Basic Sciences, Faculty of Veterinary Medicine, University of Agriculture in Krakow, Redzina 1c, 30-248 Krakow, Poland; anna.galuszka@urk.edu.pl; 2 Department of Infectious Diseases and Public Health, University of Agriculture in Krakow, 30-248 Krakow, Poland; izabela.sieminska@urk.edu.pl (I.S.); ewa.oclon@urk.edu.pl (E.O.); 3 Department of Clinical Immunology, Institute of Paediatrics, Faculty of Medicine, Jagiellonian University Medical College, Wielicka 265, 30-663 Krakow, Poland; adrianna.galuszka@uj.edu.pl; 4 Laboratory of Recombinant Proteins Production, University of Agriculture in Krakow, 30-248 Krakow, Poland

**Keywords:** telocytes, testicular interstitium, cell morphology, primary culture

## Abstract

**Highlights:**

**What are the main findings?**

- A flow cytometry-assisted enrichment strategy combining CD34-positive/CD31-negative selection with an empirical αSMA-associated exclusion step was established for rat testicular telocyte cultures.
- The telocyte-enriched culture showed recurring in vitro morphological patterns, including stellate, bipolar, branched, chain-like, and network-forming patterns.

**What are the implications of the main findings?**

- The proposed enrichment approach provides a practical experimental platform for further phenotypic, ultrastructural, molecular, and functional studies of rat testicular telocytes.
- The observed morphological patterns should be interpreted as culture-associated phenotypes and not as evidence of stable or functionally distinct telocyte subpopulations in vivo.

**Abstract:**

Telocytes are stromal cells characterized by small cell bodies and long cytoplasmic extensions termed telopodes. Although identified in the rat testicular interstitium, no standardized protocol for their isolation and in vitro enrichment has been established. This study aimed to develop an enriched primary culture model for rat testicular telocytes and characterize their morphology in vitro. Testicular cells from adult male Wistar rats were isolated by enzymatic digestion, subjected to short-term culture with differential adhesion, and enriched by fluorescence-activated cell sorting based on a CD34-positive/CD31-negative profile with empirical exclusion of events within the αSMA-associated region to reduce PTMC-like contamination. Post-sorting CD34 immunoreactivity, absence of detectable CD31 immunoreactivity, and characteristic morphology supported interpretation of the sorted fraction as telocyte-enriched. Light microscopy revealed recurring stellate, bipolar, compact branched, chain-like, and dense network-forming patterns. Some long, thin cytoplasmic processes showed a moniliform appearance with alternating thin segments and focal dilations. The findings demonstrate culture-associated morphological heterogeneity within the enriched population and provide a methodological framework for its isolation and light-microscopic characterization. Expanded post-sorting phenotyping, including PDGFRα and vimentin, together with ultrastructural validation, will be required in future studies.

## 1. Introduction

Spermatogenesis and steroidogenesis depend on precise intercellular communication within the testicular microenvironment, including the seminiferous epithelium and the interstitial compartment [1,2,3,4]. These processes require coordinated interactions among multiple cell populations, including Sertoli cells, Leydig cells, peritubular myoid cells (PTMCs), and interstitial stromal cells [1,2,3,4]. The structural and functional integrity of this multicellular system is essential for maintaining spermatogenic efficiency and hormonal balance. Among interstitial cell populations, telocytes (TCs) have emerged as a distinct stromal cell type characterized by small cell bodies and extremely long, thin cytoplasmic extensions termed telopodes [5]. These prolongations form complex three-dimensional networks and establish intercellular contacts with various neighboring cells, suggesting a role in tissue organization and intercellular communication [6]. Telocytes have been described in numerous organs, where they are implicated in tissue homeostasis, regeneration, and paracrine signaling [7]. In reproductive tissues, telocytes have been shown to establish complex structural relationships with neighboring stromal, vascular, smooth muscle, immune, and glandular elements, supporting the concept that they contribute to local tissue organization and intercellular communication [8,9]. In the testis, their strategic localization within the interstitial compartment, in proximity to Leydig cells, blood vessels, and seminiferous tubules, indicates a potential involvement in the regulation of local microenvironmental dynamics [10,11]. Similar observations in other rodent species indicate that testicular telocytes may form three-dimensional networks around interstitial cells and may participate in the structural and metabolic organization of the testicular interstitium [12]. Recent in vivo studies have further demonstrated that testicular telocyte morphology and spatial organization may vary according to anatomical compartment, developmental stage, and sexual maturation, as shown in Tibetan sheep, goats examined at different postnatal ages, and Small-tailed Han sheep before and after sexual maturation [13,14,15].

Among the stromal cell populations of the testicular interstitium, PTMCs represent a well-characterized population of contractile cells surrounding seminiferous tubules [3,16,17]. Through their smooth muscle-like properties, they contribute to sperm transport, seminiferous tubule integrity, and the modulation of Sertoli and Leydig cell function via paracrine mechanisms [3,16,17]. Their partial overlap with telocytes in stromal localization, however, may complicate the distinction between these two cell populations, particularly in vitro [10,11,16,17,18]. The identification of telocytes relies primarily on morphological criteria combined with immunophenotypic profiling. Typical features include a small cell body and the presence of long, moniliform telopodes, composed of alternating thin segments (podomers) and dilated regions (podoms) [5,6,19]. Immunohistochemically, telocytes are most commonly characterized by CD34 expression and the absence of endothelial (CD31) and myofibroblastic/contractile (α-SMA) markers, although marker expression may vary depending on tissue context [18,20,21,22]. In the rat testis specifically, telocytes have been shown to co-express CD34 and PDGFRα while remaining negative for both α-SMA and vimentin [18]. Nevertheless, overlap with other interstitial cell types, including fibroblasts and PTMCs, remains a significant challenge for their unambiguous identification [16,17,18].

Although the presence of telocytes in the testis has been increasingly documented across species, their precise role in testicular physiology remains incompletely understood [10,11,12,18,23,24,25,26]. Recent reviews have further highlighted their potential involvement in stromal organization, intercellular communication, regulation of the testicular microenvironment, and spermatogenesis, while emphasizing that many of these proposed functions still require experimental validation [24,25,26]. In different tissues, proposed functions of telocytes include maintenance of tissue architecture and participation in intercellular signaling through direct contacts and extracellular vesicle release [7,27,28,29].

In the male gonad, their localization suggests additional roles in the organization of the interstitial microenvironment and possible involvement in the regulation of steroidogenic and paracrine activity [10,11,12,18,24,25,26]. However, the lack of standardized and reproducible methods for isolating telocytes from testicular tissue has significantly hindered functional and mechanistic studies. Current approaches for testicular cell isolation are primarily based on enzymatic digestion and differential adhesion or density gradient separation, which are insufficient to achieve high-purity populations of closely related interstitial cells [30,31,32]. This limitation is particularly relevant for telocytes, whose morphological similarity to fibroblast-like and PTMC-like cells complicates their downstream analysis. To our knowledge, no standardized protocol for the isolation and enrichment of testicular telocytes has been previously described.

An additional unresolved issue concerns the potential heterogeneity of telocytes within a given tissue. While their characteristic morphology is widely recognized, variations in cell shape, number of telopodes, branching patterns, and degree of segmentation have been observed, suggesting the existence of distinct morphological or functional subtypes [5,6,7,33,34]. Moreover, telopode dynamics in vitro may be influenced by culture conditions and extracellular matrix composition, indicating that telocyte morphology can be highly responsive to the local microenvironment [33,34]. However, this heterogeneity has not been systematically described in the context of the testis, particularly under in vitro conditions. Therefore, the aim of the present study was to isolate and enrich telocytes from the rat testis using an optimized enzymatic, culture-based, and flow cytometry-assisted protocol, and to perform a detailed morphological characterization of the isolated cells under in vitro conditions. Accordingly, the morphological patterns observed in the present culture model were interpreted as in vitro phenotypes rather than predefined or functionally distinct telocyte subpopulations in vivo.

## 2. Materials and Methods

### 2.1. Animals and Tissue Collection

Testicular tissue was obtained from adult male Wistar rats (Rattus norvegicus domestica; *n* = 20), housed under standard laboratory conditions (12 h light/12 h dark cycle, 22 ± 2 °C, with food and water available ad libitum). The animals were surplus males from the institutional breeding colony and were not subjected to any study-specific experimental procedures before euthanasia.

In accordance with Article 2(1)(6) of the Polish Act of 15 January 2015 on the Protection of Animals Used for Scientific or Educational Purposes (consolidated text: Journal of Laws of 2023, item 465, as amended), killing an animal solely to obtain organs or tissues for scientific purposes is not classified as a procedure. Consequently, tissue collection for the present study did not require separate project authorization from a Local Ethics Committee for Animal Experiments.

Euthanasia was performed by trained personnel in the institutional animal facility. Deep anesthesia was induced with 4–5% isoflurane and maintained at 1–2% until complete loss of consciousness and absence of responses to external stimuli. This was followed by transection of the spinal cord under deep anesthesia. Death was confirmed before tissue collection, and the testes were collected immediately under sterile conditions.

The 3Rs principles were considered throughout the study. Replacement was considered by limiting the use of live animals to tissue collection and conducting all subsequent isolation, culture, sorting, immunofluorescence, and morphological analyses ex vivo; primary testicular tissue was required because no established non-animal model or cell line adequately reproduces the testicular stromal compartment necessary to achieve the objectives of the study. Reduction was addressed by using surplus animals already available within the institutional breeding colony, thereby avoiding the breeding or purchase of an additional group specifically for this study. Refinement was implemented by performing no study-specific interventions before euthanasia and by conducting euthanasia under deep inhalational anesthesia by trained personnel, with confirmation of death before tissue collection.

### 2.2. Testicular Cell Isolation and Primary Culture

Following euthanasia, a midline abdominal incision was performed, and the testes were carefully excised using sterile instruments. Adherent tissues, including the epididymis and surrounding connective tissue, were removed to minimize contamination. The isolated testes were immediately transferred into chilled Hank’s Balanced Salt Solution (HBSS, Thermo Fisher Scientific, Waltham, MA, USA, Cat. No. 14025092) supplemented with 100 U/mL penicillin and 100 μg/mL streptomycin (Thermo Fisher Scientific, Waltham, MA, USA, Cat. No. 15140122) and 0.01 mM HEPES (Thermo Fisher Scientific, Waltham, MA, USA, Cat. No. 15630106). The tunica albuginea and visible blood vessels were removed under sterile conditions. The tissue was thoroughly washed to eliminate residual blood and debris and then mechanically minced into approximately 1 mm^3^ fragments to increase the efficiency of enzymatic digestion.

Tissue fragments were transferred into 50 mL Falcon tubes containing DMEM/F12 medium (Gibco, Thermo Fisher Scientific, Waltham, MA, USA, Cat. No. 11320033) supplemented with collagenase type II (Thermo Fisher Scientific, Waltham, MA, USA, Cat. No. 17101015) at a concentration of 0.25 mg/mL and incubated at 37 °C for 35 min under gentle agitation to facilitate extracellular matrix degradation and release of interstitial cells. Enzymatic activity was subsequently stopped by adding chilled HBSS, and the resulting suspension was filtered sequentially through 100 μm and 40 μm cell strainers to remove undigested tissue fragments. The filtrate was centrifuged at 1000 rpm for 5 min at 4 °C, and the cell pellet was resuspended in DMEM/F12 supplemented with 10% fetal bovine serum (FBS; Gibco, Thermo Fisher Scientific, Waltham, MA, USA, Cat. No. 10091148), 100 U/mL penicillin, and 100 μg/mL streptomycin.

Isolated cells were seeded onto standard tissue culture dishes and maintained in DMEM/F12 supplemented with 10% FBS, 100 U/mL penicillin, and 100 μg/mL streptomycin. Cultures were incubated at 37 °C in a humidified atmosphere containing 5% CO_2_. To reduce the contribution of rapidly adhering fibroblast-like cells, the culture medium containing non-adherent cells was collected after 2 h of incubation, centrifuged, and the resulting pellet was resuspended in fresh culture medium. Cells were then cultured for 72 h to obtain sufficient confluency for downstream flow cytometric analysis and sorting.

### 2.3. Flow Cytometry and Cell Sorting

After 72 h of culture, cells were detached, filtered through a 40 μm cell strainer, and resuspended in FACS buffer. Prior to sorting, cell viability was assessed using the Trypan Blue exclusion assay and exceeded 99%. Cells were stained with fluorochrome-conjugated monoclonal antibodies against CD34 (NeoBiotechnologies, Union City, CA, USA, Cat. No. 947-MSM1-CF488-100T, Clone: ICO-115), CD31 (Invitrogen, Carlsbad, CA, USA, Cat. No. 25-0310-82, Clone: TLD-3A12), and α-smooth muscle actin (αSMA; Invitrogen, Carlsbad, CA, USA, Cat. No. 50-9760-82, Clone: 1A4). Fluorescence-minus-one (FMO) controls were used to establish gating thresholds for each marker and to minimize the effects of nonspecific staining and autofluorescence. Cell sorting was performed using a FACSAria II cell sorter (BD Biosciences, San Jose, CA, USA). Flow cytometry reporting and analysis were performed in accordance with established flow cytometry guidelines [35,36].

**Figure 1:**
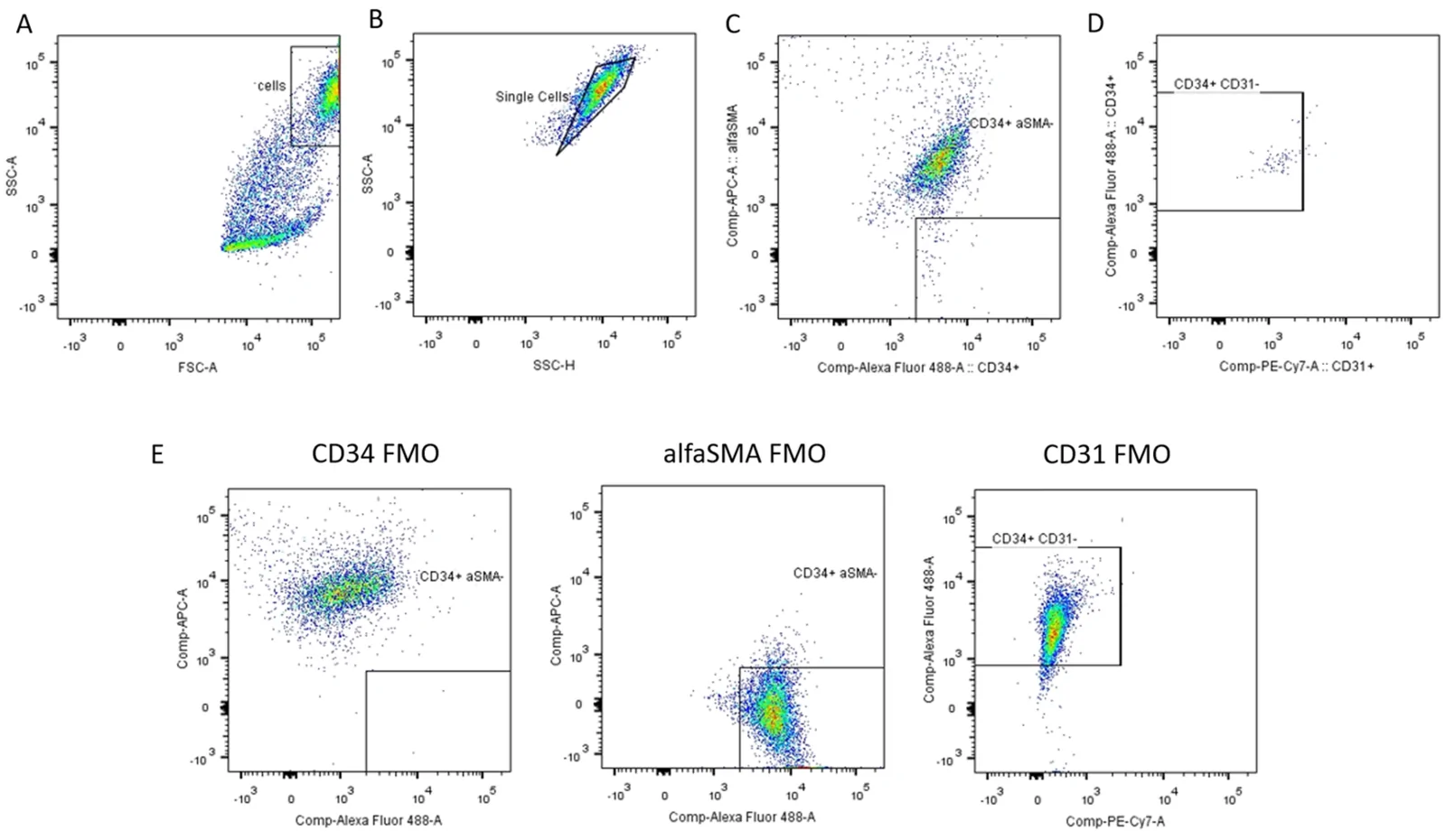
Flow cytometry gating strategy for the isolation of rat testicular telocytes. Representative flow cytometry plots from one of five independent isolation and sorting experiments showing the sequential gating strategy used to obtain a telocyte-enriched population from primary rat testicular cell cultures. Colors in the pseudocolor plots indicate relative event density, with blue representing lower and red higher event density. (**A**) Initial selection of cells based on forward scatter area (FSC-A) and side scatter area (SSC-A) to exclude debris and non-cellular events. (**B**) Identification of single cells using SSC-A versus SSC-H parameters to exclude cell aggregates and doublets. (**C**) Selection of CD34-positive cells followed by empirical exclusion of events within the αSMA-associated fluorescence region, introduced as an operational step to reduce PTMC-like/myoid stromal contamination. (**D**) Subsequent exclusion of CD31-positive endothelial cells, resulting in a CD34-positive/CD31-negative fraction interpreted as telocyte-enriched following αSMA-associated exclusion. (**E**) Representative fluorescence-minus-one (FMO) controls for CD34, αSMA-associated fluorescence, and CD31 used to establish the corresponding gating thresholds and minimize the contribution of nonspecific fluorescence and autofluorescence. The αSMA-associated exclusion gate was used solely as an empirical operational depletion step and should not be interpreted as demonstrating validated intracellular αSMA detection or an αSMA-negative telocyte phenotype.

The gating strategy is shown in Figure 1. Debris was excluded based on forward and side scatter characteristics, followed by selection of single cells using SSC-A versus SSC-H parameters. Within the single-cell population, CD34-positive cells were first identified. Events within the αSMA-associated region were then excluded as an empirical operational step intended to reduce PTMC-like/myoid stromal contamination, and CD31-positive endothelial cells were subsequently excluded using gates established from FMO controls. The collected fraction was therefore defined as a CD34-positive/CD31-negative telocyte-enriched fraction obtained following αSMA-associated exclusion and was subjected to post-sorting immunofluorescence and morphological analysis. Because no permeabilized versus non-permeabilized αSMA staining comparison was performed for the sorting protocol, αSMA status was not used as an independent criterion for cell identity. Five independent isolation and sorting experiments were performed using testicular tissue obtained from a total of 20 rats. Following sorting, the CD34-positive/CD31-negative telocyte-enriched fraction obtained after αSMA-associated exclusion was maintained in culture and monitored primarily for morphological evaluation. The study was not designed to determine the maximum duration of post-sorting culture, and the absolute number of collected cells per isolation, testis, or animal was not systematically recorded.

The inclusion of the αSMA-associated exclusion step was based on preliminary protocol optimization. Cultures obtained after CD34-positive/CD31-negative sorting alone were frequently dominated by large, flattened cells with a contractile, myoid-like morphology. Conventional αSMA immunostaining performed on fixed preliminary cultures supported the interpretation of these cells as PTMC-like contractile stromal cells. These preliminary observations were used only to guide protocol optimization and were not designed as a quantitative comparison of culture composition before and after αSMA-associated exclusion. The αSMA-associated gate was therefore introduced as a pragmatic empirical exclusion step aimed at reducing PTMC-like contamination and facilitating morphological evaluation of the telocyte-enriched fraction. The choice of αSMA was additionally informed by published studies and manufacturer-provided application information concerning its use in flow cytometric workflows [37].

Because αSMA is an intracellular cytoskeletal protein and staining during sorting was performed without permeabilization, the αSMA-associated fluorescence was not interpreted as validated intracellular αSMA detection or as a definitive phenotypic characteristic of the collected cells. Because neither a permeabilized versus non-permeabilized αSMA staining comparison nor a matched flow-cytometric PTMC-positive control was included, the biological specificity of the αSMA-associated signal under the applied sorting conditions could not be established. Instead, the αSMA-associated region was used solely as an empirical operational exclusion gate intended to reduce PTMC-like contamination. Cell identity was not assigned on the basis of αSMA status.

### 2.4. Morphological Analysis

Morphological evaluation was performed using light microscopy (Leica DM IL LED, Leica Microsystems CMS GmbH, Wetzlar, Germany) after culture and sorting. Particular attention was paid to cells displaying morphological features consistent with telocytes, including small cell bodies and long, thin cytoplasmic processes. Telocytes were identified based on established morphological criteria [5,6,18,19], including the presence of elongated telopodes with uneven thickness and occasional moniliform appearance, as well as the ability to form intercellular contacts and network-like arrangements. Cells lacking these features, such as large spindle-shaped cells with broad cytoplasmic processes consistent with peritubular myoid or fibroblast-like cells, were treated as background and excluded from detailed analysis. To assess intra-population variability, telocytes were qualitatively analyzed for differences in cell body shape, number and length of cytoplasmic processes, degree of branching, and the presence of focal dilations along the processes. Morphological analysis was performed qualitatively due to the descriptive nature of the study; systematic morphometric quantification will be addressed in future work. Representative images were acquired using an inverted light microscope (Leica DM IL LED, Leica Microsystems CMS GmbH, Wetzlar, Germany) equipped with a digital imaging system, and scale calibration was performed using a stage micrometer.

### 2.5. Immunofluorescence Staining of Cultured Cells

Cells were cultured in chamber slides (SPL Life Sciences Co., Ltd., Pocheon-si, Gyeonggi-do, Republic of Korea, Cat. No. S30108) until the desired confluency was reached. The cells were then washed in PBS and fixed with 10% neutral buffered formalin for 10 min at room temperature. Permeabilization was subsequently performed using 0.1% Triton X-100 (Sigma-Aldrich, St. Louis, MO, USA, cat. no. X100). Non-specific binding sites were blocked with 5% bovine serum albumin (BSA; Sigma-Aldrich, St. Louis, MO, USA, cat. no. A7906-10g) for 30 min. The cells were incubated with primary antibodies against CD34 (Invitrogen, Carlsbad, CA, USA, cat. no. MA5-32059, clone SI16-01; 1:25) and CD31 (Invitrogen, Carlsbad, CA, USA, cat. no. MA5-13188, clone JC/70A; 1:25) overnight at 4 °C. After washing in TBS, the cells were incubated with the appropriate species-specific secondary antibodies: Alexa Fluor^®^ 488 AffiniPure^®^ Goat Anti-Rabbit IgG, F(ab’)_2_ fragment specific (Jackson ImmunoResearch Laboratories, West Grove, PA, USA, code 111-545-006; 1:300) and Alexa Fluor^®^ 647 AffiniPure^®^ F(ab’)_2_ Fragment Donkey Anti-Mouse IgG (H + L) (Jackson ImmunoResearch Laboratories, West Grove, PA, USA, code 715-606-150; 1:300) for 60 min in the dark. Before mounting, TrueVIEW reagent was applied, and the preparations were mounted in a DAPI-containing mounting medium (Vector Laboratories, Newark, CA, USA, cat. no. SP-8500). Fluorescence images were acquired using a Leica, DM2500 equipped with appropriate fluorescence filter sets, and images were processed using ImageJ version 1.53t (National Institutes of Health, Bethesda, MD, USA). Negative controls included omission of primary antibodies/single-stained controls, and identical acquisition settings were used for all samples compared.

## 3. Results

### 3.1. Isolation and Enrichment of a CD34^+^CD31^−^ Telocyte-Enriched Testicular Cell Fraction Following αSMA-Associated Exclusion

Following enzymatic digestion and short-term primary culture, adherent interstitial cells were obtained from rat testicular tissue. After 72 h of culture, the cells reached sufficient confluency for flow cytometric analysis and sorting. The gating strategy included the initial exclusion of debris based on forward scatter and side scatter parameters, followed by the selection of single cells using SSC-A versus SSC-H characteristics (Figure 1A,B). Within the single-cell population, CD34-positive cells were first selected. Events within the αSMA-associated region were then excluded as an empirical operational step intended to reduce PTMC-like contamination, and CD31-positive endothelial cells were subsequently excluded (Figure 1C,D). This strategy yielded a CD34-positive/CD31-negative cell fraction following αSMA-associated exclusion. The collected cells were subsequently evaluated by post-sorting immunofluorescence and morphological analysis and were interpreted as a telocyte-enriched population. The empirical αSMA-associated exclusion step was introduced to reduce PTMC-like contamination and to facilitate subsequent morphological assessment of cells displaying small cell bodies and long, thin cytoplasmic processes consistent with telocyte morphology. Fluorescence-minus-one controls for CD34, αSMA, and CD31 were used to define gating thresholds and to minimize nonspecific staining and autofluorescence (Figure 1E).

During preliminary protocol optimization, cultures obtained after CD34-positive/CD31-negative sorting alone were frequently dominated by large, flattened cells with a contractile, myoid-like morphology. Conventional αSMA immunostaining performed on fixed preliminary cultures supported the interpretation of these cells as PTMC-like contractile stromal cells. This observation provided the practical rationale for introducing an empirical αSMA-associated exclusion step into the flow cytometry strategy. These preliminary observations were used to guide protocol optimization and were not designed as a quantitative comparison of culture composition before and after αSMA-associated exclusion.

**Figure 2:**
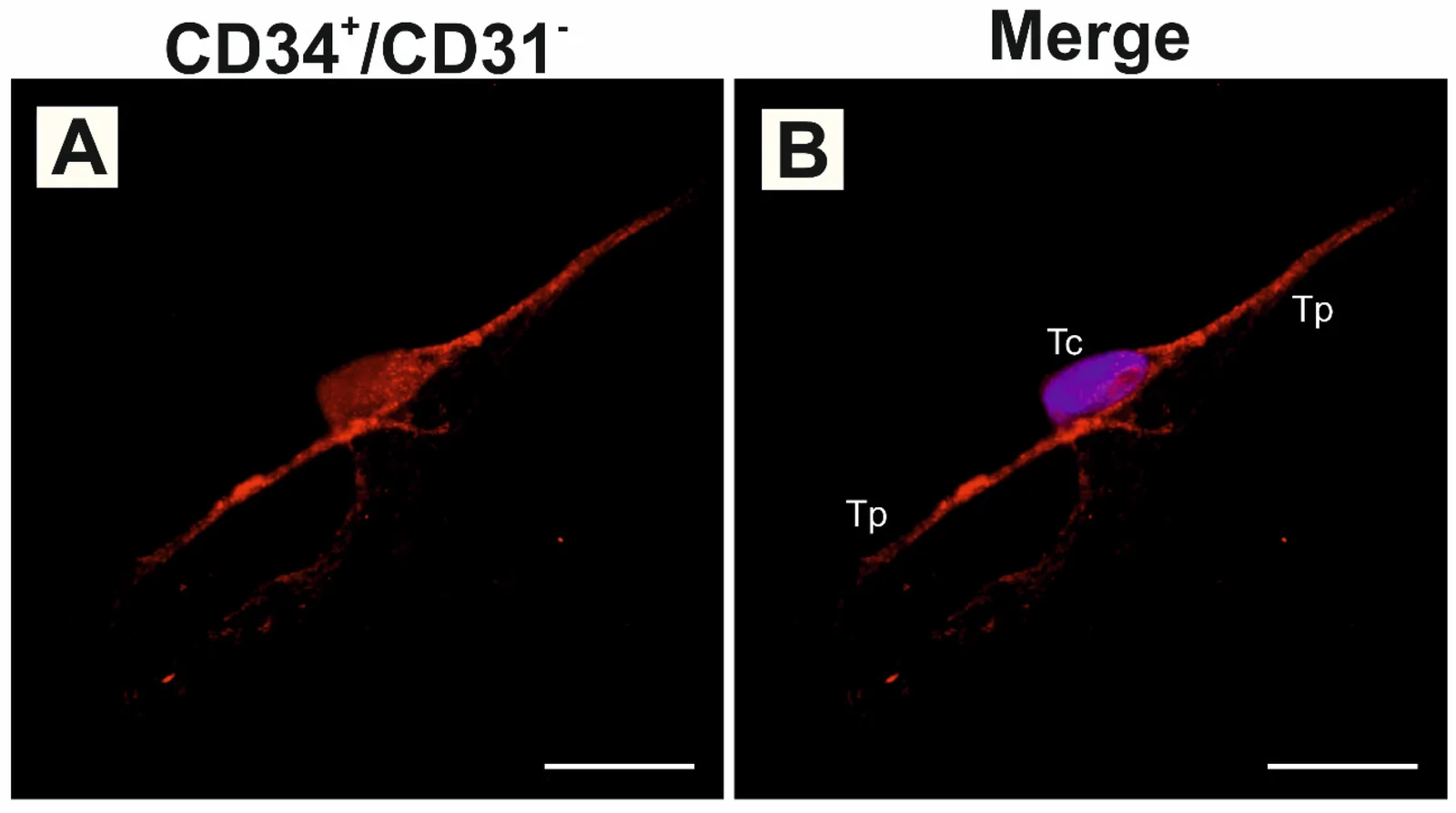
CD34 expression in the telocyte-enriched rat testicular cell fraction. Representative immunofluorescence images showing cultured cells after flow cytometric enrichment. (**A**) CD34 immunoreactivity is visible in the telocyte cell body and along long, thin telopodes. (**B**) Merged image showing CD34-positive cytoplasmic staining and nuclear counterstaining with DAPI, showing a small cell body and elongated cytoplasmic processes consistent with telocyte morphology. Red fluorescence indicates CD34 immunoreactivity, while blue fluorescence represents DAPI nuclear counterstaining. CD31 signal was absent in these cells, supporting their non-endothelial phenotype. Overall, the combined CD34-positive/CD31-negative profile and characteristic morphology support the interpretation of the isolated cells as a telocyte-enriched population. Abbreviations: Tc, telocyte cell body; Tp, telopode. Scale bars: 20 µm.

To further support the identity of the isolated cells, immunofluorescence analysis was performed on cultured sorted cells. CD34 immunoreactivity was detected in cells displaying a small cell body and long, thin cytoplasmic processes, including signal within the cell body and along telopodes (Figure 2A). In contrast, CD31 immunoreactivity was not detected in these cells, supporting their non-endothelial phenotype. Merged images with DAPI nuclear counterstaining showed that CD34-positive cells displayed small cell bodies and elongated cytoplasmic processes consistent with telocyte morphology (Figure 2B). Taken together, the CD34-positive/CD31-negative profile, post-sorting CD34 immunoreactivity, absence of detectable CD31 immunoreactivity, and characteristic morphology supported the interpretation of the sorted cells as a telocyte-enriched population.

### 3.2. Morphological Characteristics of Cultured Rat Testicular Telocytes

**Figure 3:**
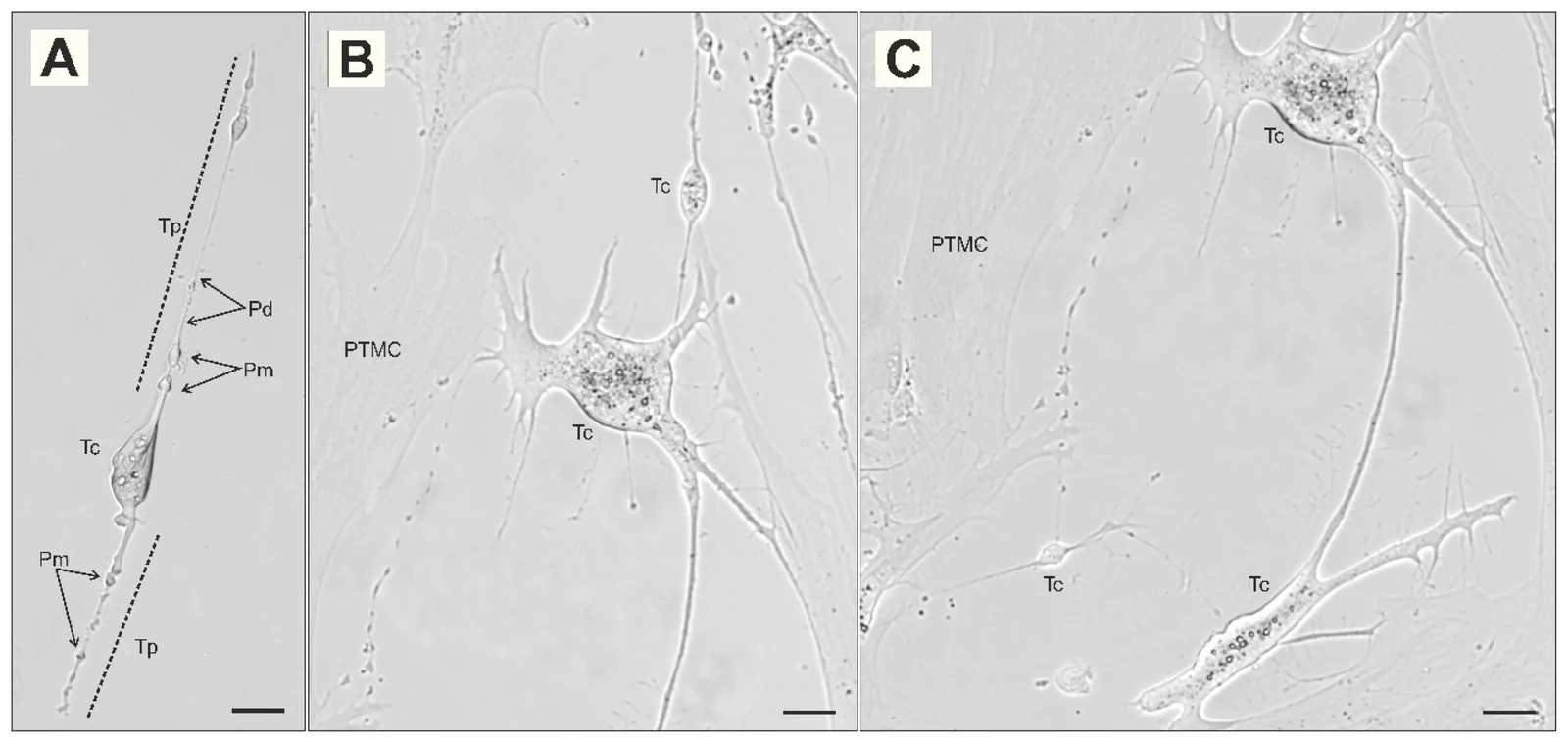
General morphology of telocytes isolated from rat testicular tissue and cultured in vitro. Representative inverted light microscopy images showing telocytes with small cell bodies and long, thin cytoplasmic processes. (**A**) A single telocyte showing a small cell body and two long telopodes. The processes display an uneven caliber, with alternating thin segments and focal dilations producing a moniliform appearance. (**B**,**C**) Telocytes observed in the vicinity of larger peritubular myoid cell-like stromal cells. Telocytes are characterized by small oval or piriform cell bodies and long, delicate cytoplasmic processes, whereas the larger stromal cells display broader cytoplasm and lack long moniliform telopodes. Scale bars: 20 µm. Abbreviations: Tc, telocyte; Tp, telopode; Pd, podomer; Pm, podom; PTMC, peritubular myoid cell-like stromal cell.

Following flow cytometric sorting, the CD34-positive/CD31-negative telocyte-enriched fraction obtained after αSMA-associated exclusion was plated and allowed to adhere, and morphological evaluation was initiated after 24 h. Following sorting, the cells adhered and retained the morphological features evaluated in the present study, including small cell bodies, long, thin telopodes, branching, and network-like arrangements. No appreciable increase in cell number was observed during the post-sorting observation period, suggesting limited apparent expansion under the applied culture conditions. Because proliferation was not assessed using dedicated proliferation markers, cell-cycle analysis, or longitudinal quantitative cell counting, no definitive conclusions regarding proliferative capacity were drawn. Cultured telocytes were characterized by small oval, piriform, elongated, or occasionally stellate cell bodies and by long, thin cytoplasmic processes corresponding to telopodes (Figure 3A–C). In the majority of cells, these processes extended over several cell body lengths and displayed a delicate, filamentous appearance. In a subset of cells, the telopodes showed uneven caliber, with alternating thin segments and focal dilations producing a moniliform appearance at the light-microscopic level (Figure 3A). These features were consistent with morphological criteria previously described for telocytes in other tissues [5,6,19]. Larger peritubular myoid cell-like background cells were also observed in some culture fields. These cells displayed broader cytoplasm and a more flattened morphology and lacked the long, delicate, moniliform telopodes characteristic of telocytes (Figure 3B,C). This morphological contrast allowed cells displaying features consistent with telocytes to be distinguished from surrounding PTMC-like stromal cells during microscopic evaluation. Residual PTMC-like or fibroblast-like stromal cells were still occasionally observed, confirming that the procedure resulted in enrichment rather than complete purification. Because systematic low-magnification cell counting and additional post-sorting immunostaining for αSMA or vimentin were not performed, the proportion of residual non-telocyte stromal cells could not be reliably quantified.

**Figure 4:**
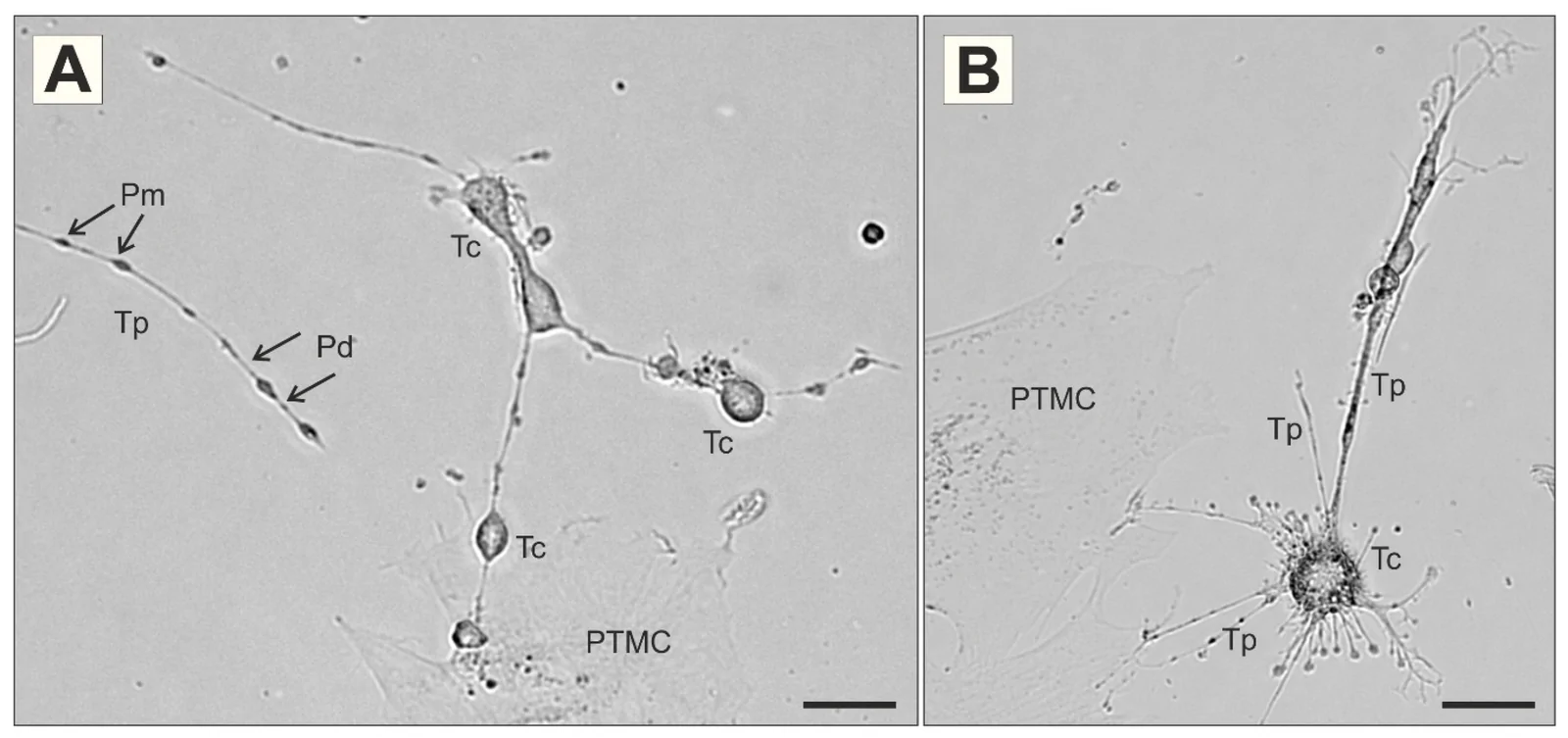
Telopodial organization and moniliform morphology of rat testicular telocytes in vitro. Representative light microscopy images illustrating telocytes with long, thin cytoplasmic processes and variable process organization. (**A**) Telocytes displaying long telopodes with alternating thin segments and focal dilations, producing a characteristic moniliform appearance. Larger peritubular myoid cell-like stromal cells are visible in the surrounding culture area. (**B**) A telocyte with a small cell body and multiple radiating telopodes, forming a stellate or hub-like morphology. Long telopodes extend from the cell body and show focal dilations along their course. Scale bars: 20 µm. Abbreviations: Tc, telocyte; Tp, telopode; Pd, podomer; Pm, podom; PTMC, peritubular myoid cell-like stromal cell.

A characteristic feature of cultured testicular telocytes was the presence of long telopodes with a moniliform organization. Unlike the more uniform cytoplasmic processes of surrounding stromal cells, telopodes frequently displayed alternating thin segments and focal dilations along their course (Figure 4A). These dilations varied in size and distribution between cells, contributing to the morphological variability observed within the telocyte population. In some cells, telopodes extended radially from a small central cell body, producing a stellate or hub-like morphology (Figure 4B).

### 3.3. Morphological Heterogeneity and Network-Forming Patterns

**Figure 5:**
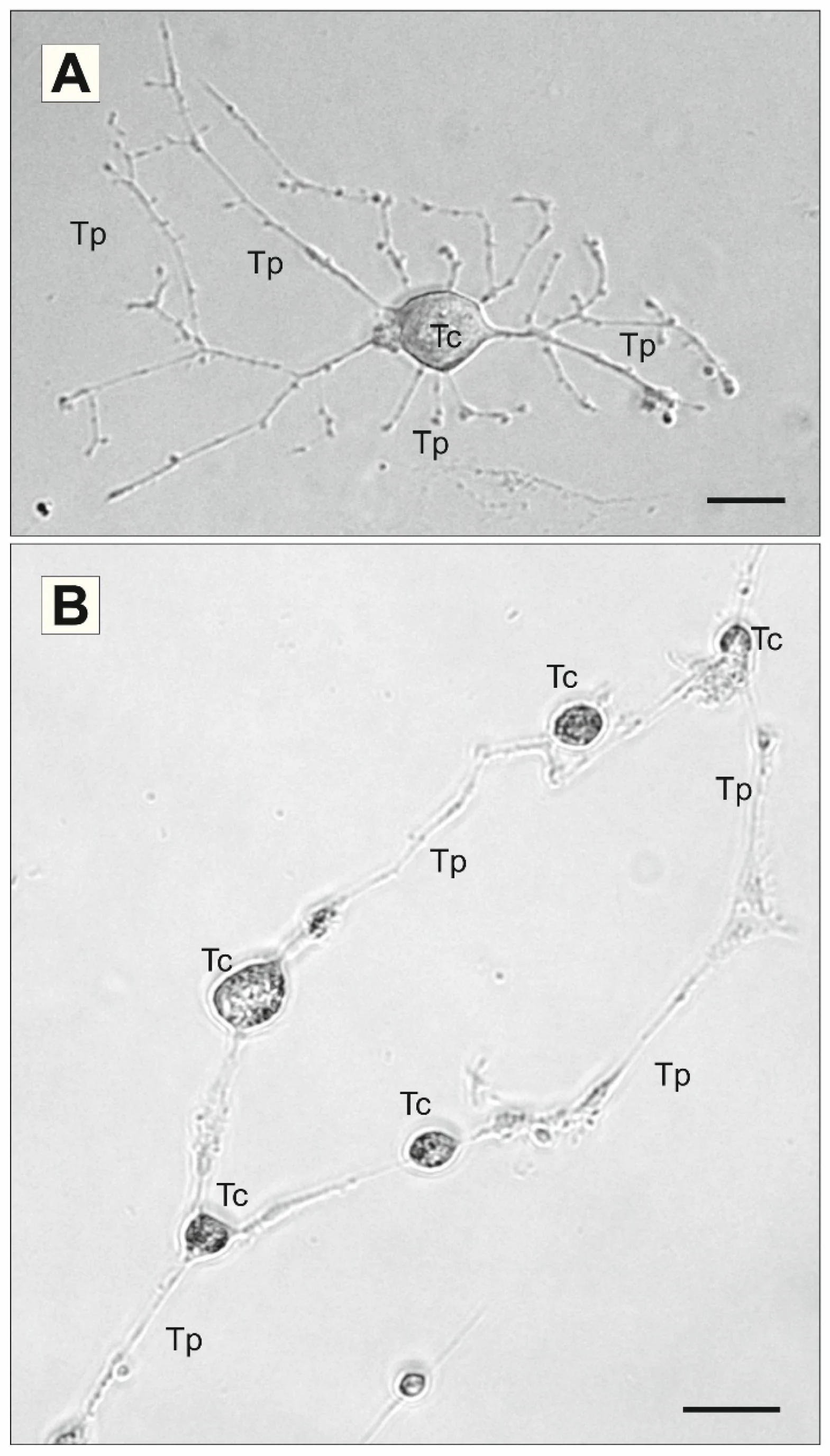
Morphological heterogeneity of rat testicular telocytes cultured in vitro. Representative inverted light microscopy images showing distinct morphological patterns of telocytes (TCs) isolated from rat testicular tissue. (**A**) A stellate, highly ramified telocyte with a small central cell body and multiple long telopodes (Tps) radiating in different directions. Several telopodes show secondary branching and focal dilations along their course. (**B**) Network-forming telocytes connected by long, thin telopodes. The cells are arranged in a chain-like network, with telopodes establishing contacts between neighboring telocyte cell bodies. Scale bars: 20 µm. Abbreviations: Tc, telocyte; Tp, telopode.

**Figure 6:**
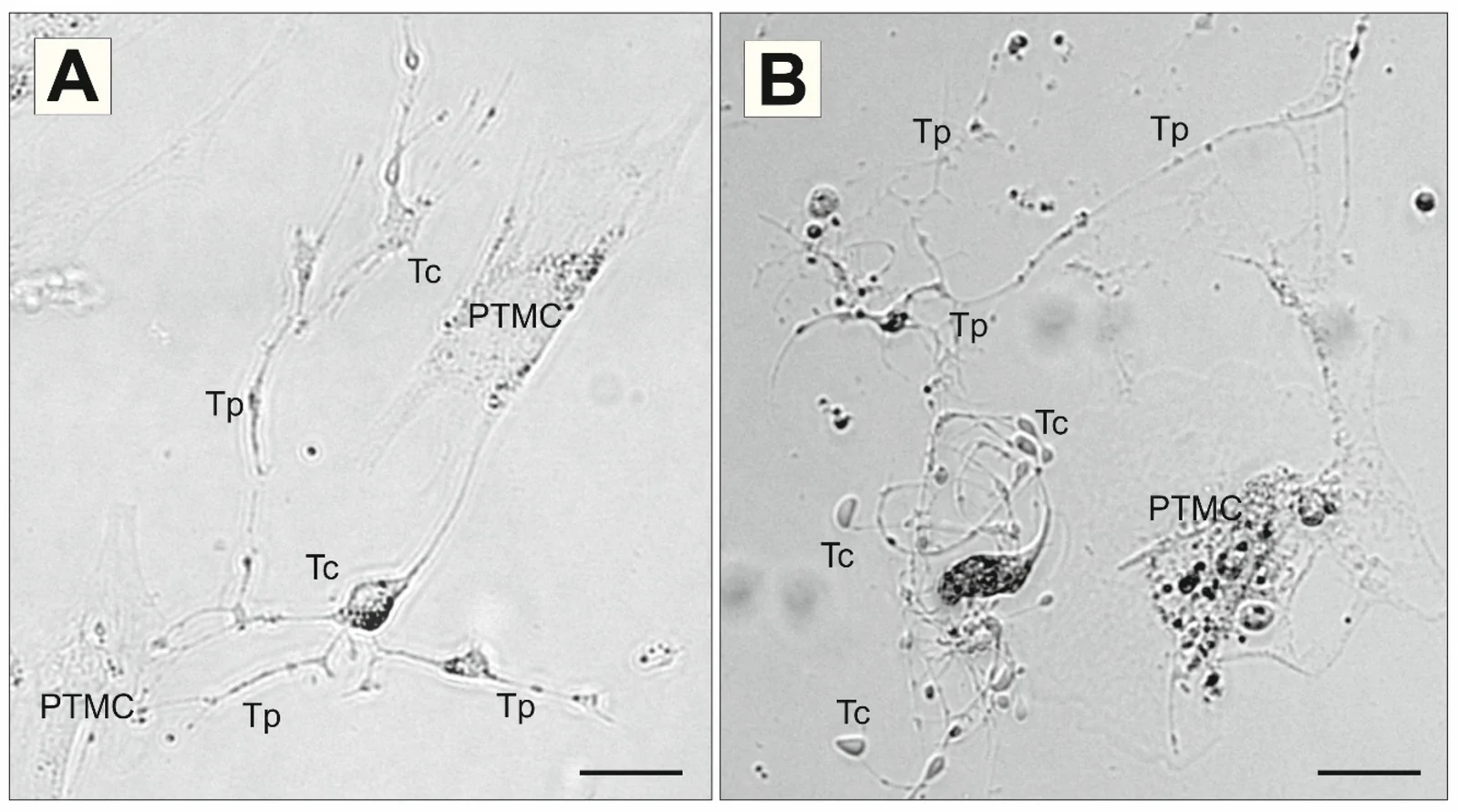
Telocytes in the vicinity of peritubular myoid cells in primary rat testicular cell culture. Representative inverted light microscopy images showing telocytes (TCs) located close to larger peritubular myoid cells (PTMCs). (**A**) Telocytes with small cell bodies and long, thin telopodes (Tps) extending between surrounding PTMCs. The telopodes show delicate, elongated morphology and occasionally uneven caliber along their course. (**B**) Telocytes forming a loose network-like arrangement in the vicinity of a larger PTMC. Telocytes are morphologically distinguishable from PTMCs by their smaller cell bodies and long, slender telopodes, whereas PTMCs display broader cytoplasm and a more flattened morphology. Scale bars: 20 µm. Abbreviations: Tc, telocyte; Tp, telopode; PTMC, peritubular myoid cell.

**Figure 7:**
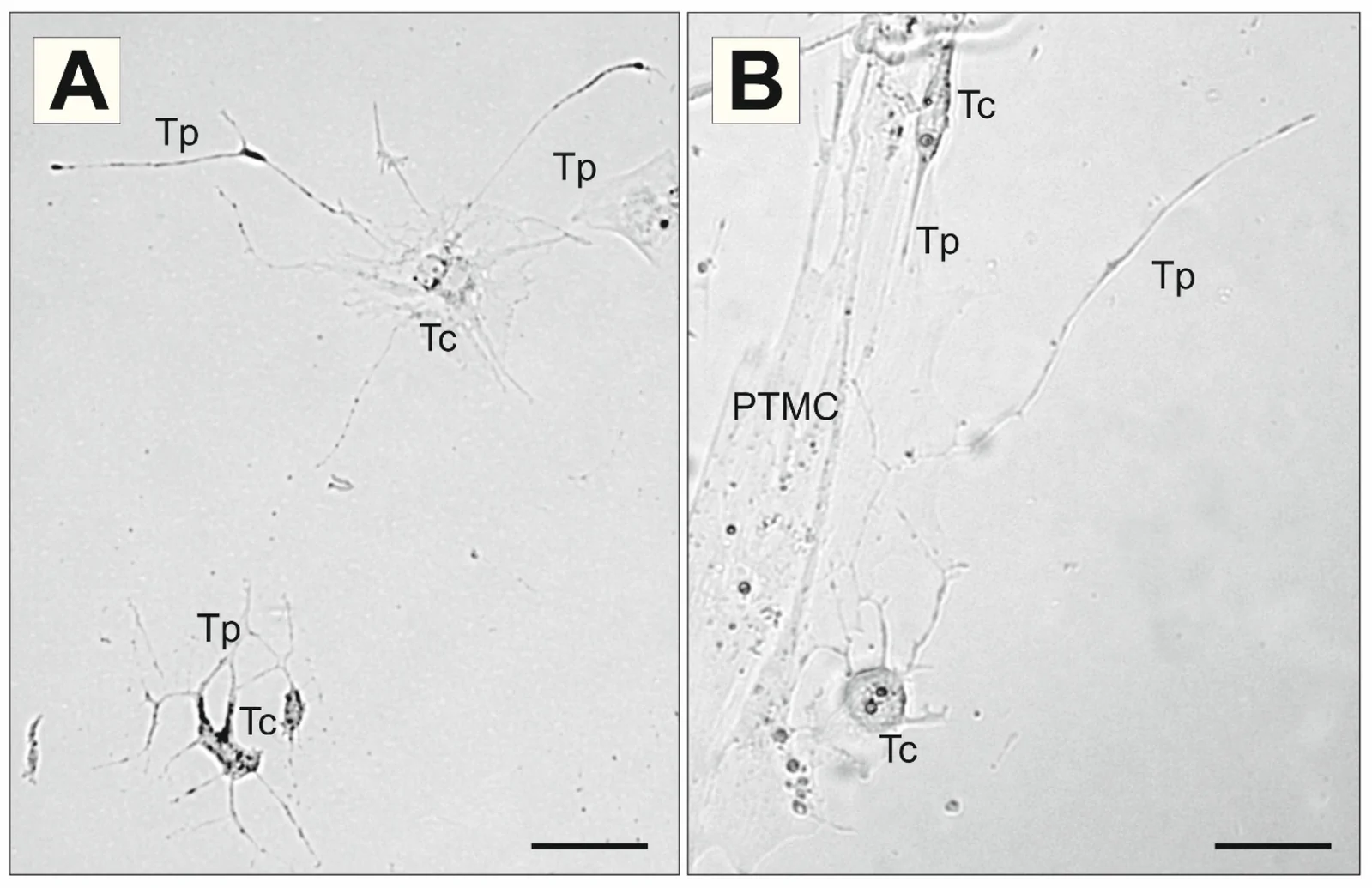
Spatial arrangement of telocytes and their telopodes in primary rat testicular cell culture. Representative inverted light microscopy images showing telocytes (TCs) with long, thin telopodes (Tps) in vitro. (**A**) Two telocytes displaying distinct morphologies within the same culture field. The upper TC shows a small cell body with very long telopodes extending over a considerable distance, whereas the lower TC displays a more compact, branched morphology with several shorter telopodes. (**B**) Telocytes located in the vicinity of a larger peritubular myoid cell (PTMC). Telopodes extend along and away from the PTMC, highlighting the morphological distinction between small, process-bearing TCs and larger PTMCs with broader cytoplasm. Scale bars: 20 µm. Abbreviations: Tc, telocyte; Tp, telopode; PTMC, peritubular myoid cell.

**Figure 8:**
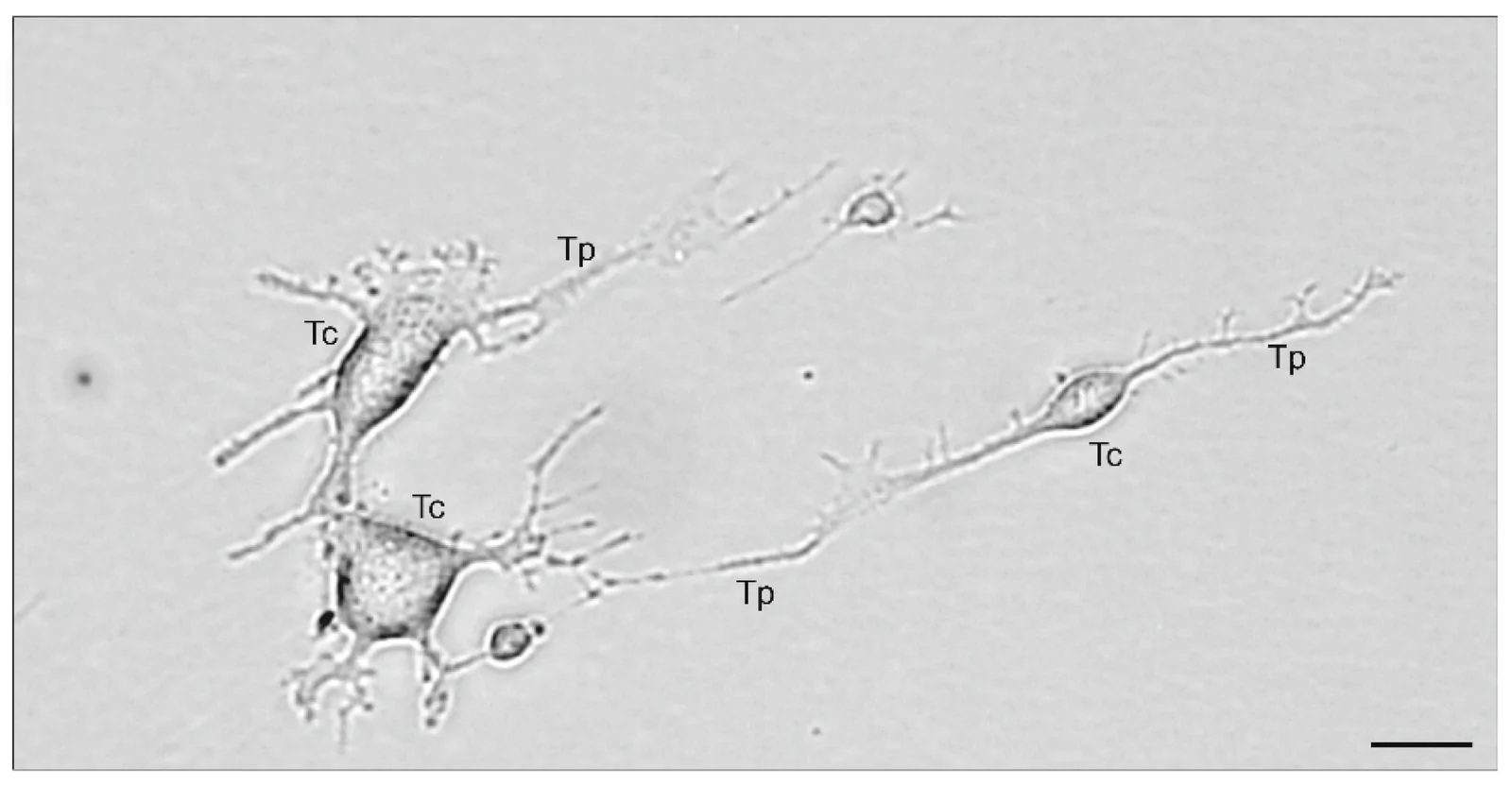
Local network-like arrangement of rat testicular telocytes in vitro. Representative inverted light microscopy image showing a small group of telocytes (TCs) connected by long, thin telopodes (Tps). Several TCs are arranged in close proximity, with telopodes extending between neighboring cell bodies and forming a loose chain-like or local network-like structure. The observed morphology illustrates the ability of cultured testicular telocytes to establish intercellular contacts through elongated cytoplasmic processes. Scale bar: 20 µm.

**Figure 9:**
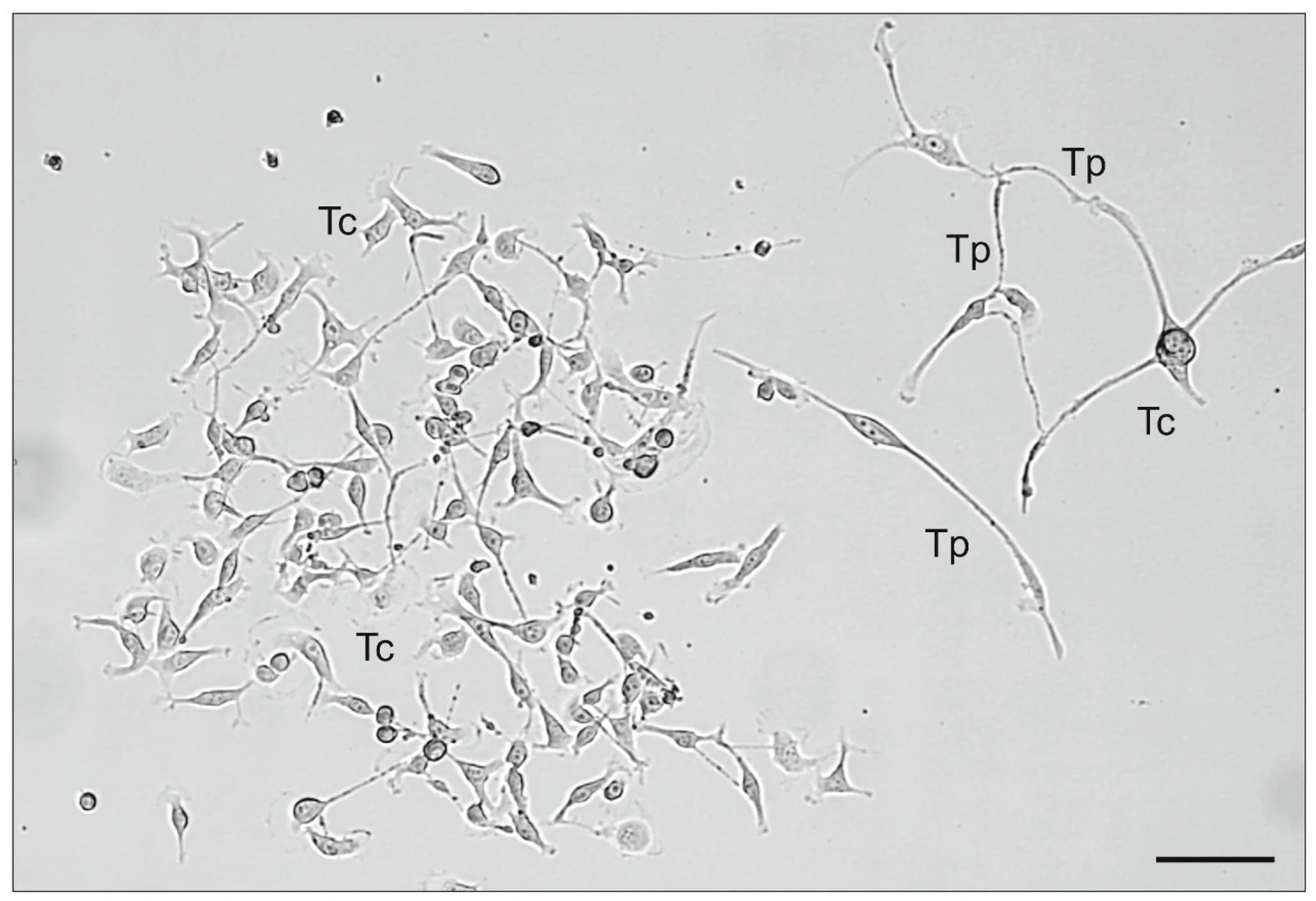
Local dense network of rat testicular telocytes cultured in vitro. Representative inverted light microscopy image showing a locally dense network of telocytes (TCs) and long, thin telopodes (Tps). On the left side of the image, numerous TCs form a compact network-like arrangement with multiple intercellular contacts. On the right side, individual TCs are connected by long telopodes extending over a considerable distance from the cell body. This image illustrates both the network-forming capacity of cultured testicular telocytes and the presence of long, delicate cytoplasmic processes linking neighboring cells. Scale bar: 40 µm. Abbreviations: Tc, telocyte; Tp, telopode.

Marked heterogeneity was observed within the telocyte population. Several recurring morphological patterns were observed, including highly ramified stellate cells, bipolar or elongated cells, compact branched cells, and network-forming cells (Figure 5, Figure 6, Figure 7, Figure 8 and Figure 9). Some telocytes exhibited a stellate or highly ramified morphology, with a small central cell body and numerous telopodes radiating in different directions (Figure 5A). These cells frequently showed secondary branching of telopodes and appeared as local organizing points within the culture. In other fields, telocytes were connected by long, thin telopodes, forming chain-like or loose network-like arrangements between neighboring cell bodies (Figure 5B). These observations indicate that telocytes may occur both as isolated cells and as interconnected cellular networks under in vitro conditions.

Telocytes were also observed in close proximity to larger PTMC-like stromal cells (Figure 6A,B). Despite this proximity, telocytes remained morphologically distinct, showing small cell bodies and long, slender telopodes extending between or around the larger cells. PTMC-like cells, in contrast, had broader cytoplasm and lacked typical long moniliform telopodes (Figure 6A,B).

Within the same culture fields, telocytes displayed different degrees of process extension and branching. Some cells showed very long, sparsely branched telopodes extending over a considerable distance, whereas others displayed a more compact morphology with several shorter, branched processes (Figure 7A). Telocytes located near PTMC-like cells extended telopodes along or away from the larger cell bodies, further emphasizing the morphological distinction between these two stromal cell types (Figure 7B).

In some areas, neighboring telocytes were arranged in close proximity and connected by long, thin telopodes, forming loose chain-like or local network-like structures (Figure 8). This pattern differed from isolated bipolar or stellate cells and suggested that telocytes can establish local intercellular arrangements through direct telopodial contacts.

The most complex organization was observed in locally dense telocyte networks. In these fields, numerous telocytes formed compact network-like arrangements with multiple intercellular contacts (Figure 9). Individual telocytes were also connected over longer distances by delicate telopodes, indicating that network formation occurred both within dense cellular clusters and between more distant cells (Figure 9). These findings support the view that cultured rat testicular telocytes represent a morphologically heterogeneous and spatially organized stromal cell population rather than a uniform cell type.

## 4. Discussion

The present study reports, to our knowledge, the first enrichment of telocytes from rat testicular tissue using a culture-based, flow cytometry-assisted isolation protocol. The resulting CD34-positive/CD31-negative telocyte-enriched fraction obtained following αSMA-associated exclusion displayed marked morphological heterogeneity under in vitro conditions. This marker profile is consistent with previous descriptions of rat testicular telocytes [18] and with broader reports on CD34-positive telocytes or telocyte-like stromal cells in other tissues [20,21,22]. At the morphological level, the isolated cells showed classical telocyte features, including small cell bodies, long telopodes, and moniliform prolongations [5,6,19]. Their chain-like and network-forming arrangements were also consistent with previous observations of telocytes maintained under in vitro culture conditions [33,34,38]. Importantly, the observed variability was not limited to minor differences in cell size but involved distinct patterns of cellular organization. Stellate and bipolar morphologies corresponded to classical descriptions of telocyte morphology [5,6], whereas compact branched, chain-like, and dense network-forming phenotypes were in line with reports describing telocyte plasticity and organization in culture [33,34,38].

The enrichment strategy used in this study was based primarily on the selection of CD34-positive cells and exclusion of CD31-positive endothelial cells. An additional αSMA-associated exclusion step was introduced pragmatically to reduce PTMC-like/myoid stromal contamination, but αSMA status was not used as an independent criterion for telocyte identification. In the rat testis, Liu et al. demonstrated that telocytes express CD34 and PDGFRα while remaining negative for αSMA and vimentin [18], supporting the relevance of a telocyte-oriented enrichment strategy while also highlighting the need for expanded post-sorting phenotyping. The subsequent detection of CD34 immunoreactivity, absence of detectable CD31 immunoreactivity, and characteristic morphology supported the interpretation of the sorted fraction as telocyte-enriched. The broader use of CD34 positivity, together with exclusion of endothelial and myofibroblastic markers, is also consistent with studies on telocytes and CD34-positive stromal cells in other tissues [20,21,22]. The use of CD31 exclusion was intended to reduce endothelial cell contamination, whereas the αSMA-associated exclusion step was introduced pragmatically to reduce the contribution of PTMC-like/myoid stromal cells [3,16,17]. It should be noted, however, that αSMA is an intracellular cytoskeletal protein, and its use as a live-cell sorting marker represents a methodological consideration that is addressed in detail in the limitations section. Fluorescence-minus-one controls were particularly important because primary testicular cultures may contain autofluorescent cells, debris, and heterogeneous stromal populations that can interfere with accurate gating of low-abundance interstitial cell subsets [35,36]. The subsequent detection of CD34 immunoreactivity in both the cell body and telopodes further supported the identification of the sorted cells as telocytes, in line with the CD34-positive phenotype previously reported in rat testicular telocytes [18] and other telocyte/CD34-positive stromal cell populations [20,21,22].

The most prominent morphological feature of the isolated cells was the presence of long, thin cytoplasmic processes corresponding to telopodes, which are regarded as the defining morphological hallmark of telocytes [5,6]. Telopodes are typically described as very long, slender prolongations with alternating thin segments and dilated regions, referred to as podomers and podoms, respectively [5,6,19]. Similar ultrastructural organization has been documented in telocytes from several tissues, including reproductive organs [8,39,40]. In the present study, many telopodes displayed uneven caliber and focal dilations, giving them a moniliform appearance consistent with the classical description of telocyte morphology [5,6]. The dilated and thin segments observed here, however, were identified at the light microscopic level and are therefore described as focal dilations and thin segments rather than definitively confirmed podoms and podomers. Although the present study was based primarily on light microscopy, the CD34-positive/CD31-negative profile, post-sorting CD34 immunofluorescence, absence of detectable CD31 immunoreactivity, and characteristic morphology support the interpretation of the collected cells as a telocyte-enriched population, while definitive ultrastructural confirmation of their processes will require transmission electron microscopy [18,19,20,21,22,38].

Several recurring morphological patterns were documented within the telocyte-enriched culture, including stellate, bipolar or elongated, compact branched, chain-like, and dense network-forming arrangements. Stellate and bipolar morphologies were consistent with classical descriptions of telocyte morphology [5,6], whereas branched, chain-like, and network-forming arrangements have also been reported in cultured telocyte populations [33,34,38,41,42]. Comparable morphological variability has been described in rat testicular tissue and in the testis and epididymis of sheep [13,18]. Network-forming arrangements have previously been discussed in the context of potential intercellular communication and stromal organization [29,34,41,42]; however, the present study did not assess these functions experimentally. Therefore, the observed patterns demonstrate morphological variability under the applied in vitro conditions but do not establish functional specialization or stable telocyte subpopulations.

Previous studies have described variation in testicular telocyte morphology and distribution according to species, anatomical location, reproductive status, and local stromal or endocrine conditions [10,11,12,18,23,24]. In Tibetan sheep, transmission electron microscopy, toluidine blue staining, immunohistochemistry, and double immunofluorescence identified telocytes near basement membranes and capillaries and demonstrated differences in their morphology and distribution between the testis and individual regions of the epididymis [13]. In goats, transmission electron microscopy and double immunofluorescence identified testicular telocytes as CD34-positive/vimentin-positive cells and revealed age-related differences between prepubertal, peripubertal, and sexually mature animals, including greater cell length, more numerous cytoplasmic processes, multilayered peritubular arrangements, and increased telopode-associated vesicle density in mature animals [14]. Together with the established description of telocyte networks in the human testis [11], these studies indicate that telocyte morphology and spatial organization in vivo may vary according to species, anatomical compartment, and developmental stage. However, these observations provide only a comparative framework and do not establish direct correspondence with the culture-associated morphological patterns documented in the present study.

The observed differences in cell shape, branching, process length, and network organization may reflect reversible culture-associated plasticity, different stages of cell attachment and spreading, or responses to local cell density and cell–cell interactions [33,34,38]. Although biologically distinct cellular states cannot be excluded, these possibilities remain hypothetical and cannot be resolved by the present descriptive study. Accordingly, the current findings should be interpreted as evidence of culture-associated morphological variability rather than proof of predefined, intrinsic, or functionally distinct telocyte subpopulations in vivo.

The morphological distinction between cells displaying features consistent with telocytes and neighboring PTMC-like stromal cells is consistent with previous descriptions of the spatial relationships between telocytes and contractile, vascular, immune, or other stromal elements in reproductive and non-reproductive tissues [8,9,40,41,43,44]. In the present study, this morphological contrast illustrates the practical value of combining flow cytometric enrichment with post-sorting microscopic assessment in complex primary testicular cultures. This methodological interpretation is consistent with previous work on rat testicular telocytes, CD34-positive stromal cell isolation, and comparative analyses distinguishing telocytes from fibroblast-like cells [18,20,44].

The absence of transmission electron microscopy represents a limitation of the present study. TEM remains the most reliable approach for ultrastructural characterization of telocytes and their telopodial components [19]. Although light microscopy allowed assessment of overall cell shape, process length, branching, intercellular arrangements, and moniliform organization, future ultrastructural studies will be required to characterize individual telopodial components in greater detail [13,19,38,39,45].

The post-sorting immunophenotypic characterization in the present study was limited to CD34 and CD31. Additional assessment of PDGFRα and vimentin would further strengthen the phenotypic characterization of the enriched population and should therefore be included in future studies [18]. Telocyte identification relies on the combined assessment of morphology and immunophenotype rather than on a single universally specific molecular marker [20,21,22]. In the present study, interpretation of the enriched population was supported by the CD34-positive/CD31-negative profile, post-sorting CD34 immunoreactivity, absence of detectable CD31 immunoreactivity, and characteristic cellular morphology.

A central methodological limitation of the present study is the use of an αSMA-associated exclusion gate during non-permeabilized live-cell sorting. The choice of αSMA was biologically and empirically supported by the myoid-like morphology and αSMA immunoreactivity of the cells overgrowing preliminary CD34-positive/CD31-negative cultures. These preliminary observations were used to guide protocol optimization but were not designed as a quantitative comparison of culture composition before and after αSMA-associated exclusion. The choice of this marker was additionally informed by published studies and manufacturer-provided application information concerning its use in flow-cytometric workflows [37]. However, conventional αSMA immunostaining of fixed preliminary cultures does not validate αSMA detection under non-permeabilized flow-cytometric conditions. Because neither a direct comparison between permeabilized and non-permeabilized cells nor a matched flow-cytometric PTMC-positive control was performed, the specificity and biological meaning of the αSMA-associated signal cannot be conclusively established. Accordingly, the αSMA-associated gate was used only as an empirical operational depletion step intended to reduce PTMC-like contamination and was not assigned independent phenotypic significance.

The interpretation of the collected population was based on the CD34-positive/CD31-negative profile, post-sorting CD34 and CD31 immunofluorescence, and morphological features consistent with telocytes, rather than on αSMA status alone. Thus, the αSMA-associated exclusion step should be regarded as a pragmatic enrichment tool rather than validated live-cell αSMA immunophenotyping or evidence of an αSMA-negative telocyte phenotype. An additional limitation is that the relative proportion of the telocyte-enriched population and residual PTMC-like or fibroblast-like stromal cells was not quantitatively assessed. The available images were acquired for representative morphological characterization rather than according to a predefined random-sampling protocol, and retrospective estimation of culture composition would therefore be potentially biased. Future studies should include quantitative low-magnification imaging and expanded post-sorting immunophenotyping to determine the degree of enrichment and the proportion of residual stromal cell populations. Another limitation is the descriptive nature of the morphological analysis, because the identified phenotypes were not quantified using systematic morphometric criteria. Our qualitative observations indicated limited apparent proliferation of the enriched cells after sorting, as no appreciable increase in cell number was observed during the post-sorting culture period. However, proliferation was not evaluated using dedicated molecular markers, cell-cycle analysis, or systematic longitudinal cell counting. This observation should therefore be interpreted cautiously and cannot be considered a quantitative assessment of proliferative capacity.

The present study did not include functional assays, and the observed morphological patterns cannot be interpreted as evidence of functionally distinct subpopulations. The study was designed as a methodological and morphological proof-of-concept aimed at developing an enrichment strategy and documenting morphological variability within the resulting culture. No assays of paracrine factor production, extracellular vesicle release, migration, cell-cycle activity, or interactions with neighboring testicular cell populations were performed.

The methodological value of the proposed approach lies in providing an enriched culture system that can be used in future studies combining expanded immunophenotyping, transmission electron microscopy, quantitative morphometry, and single-cell molecular analysis [18,19,24]. Dedicated proliferation and migration assays, secretome profiling, including evaluation of VEGF and TGF-β, and extracellular vesicle characterization will be required to determine whether the observed morphological patterns are associated with measurable functional differences. Co-culture experiments involving Leydig, peritubular myoid, endothelial, or immune cells may additionally help assess potential interactions within the testicular microenvironment [24,27,28,29]. Such studies will be necessary to determine whether the documented patterns represent reversible culture-associated plasticity or biologically meaningful stromal cell states [33,34,38].

The maximum period for which the enriched cells could be maintained in culture was not systematically evaluated. The stability of telocyte morphology and immunophenotype across serial passages was not systematically assessed. In addition, the absolute number of collected cells per isolation, testis, or animal was not recorded. Consequently, the yield, post-sorting recovery, purity, and reproducibility of cell recovery cannot be quantitatively assessed in the present study. Future protocol-validation studies should include the number of sorted events, post-sorting recovery and purity, viability, longitudinal proliferation assessment, and cell yield normalized to the amount of starting tissue. The morphological patterns documented in the present study were observed after enzymatic dissociation, differential adhesion, short-term culture, flow-cytometric enrichment, replating, and adaptation to an artificial in vitro environment. These procedures may have influenced cell shape, process extension, branching, and network formation [33,34,38]. The observed patterns should therefore be interpreted as in vitro phenotypes and cannot be assumed to represent stable or naturally occurring telocyte subpopulations within the intact testicular interstitium.

Despite these limitations, the present study establishes, to our knowledge, the first enriched primary culture model for rat testicular telocytes and provides a systematic characterization of their morphological heterogeneity under in vitro conditions. The optimized isolation strategy enabled enrichment of a CD34-positive/CD31-negative telocyte-enriched population from rat testicular tissue following αSMA-associated exclusion, whereas CD34 immunofluorescence and microscopic analysis supported the interpretation of the sorted cells as displaying features consistent with telocytes [18,20,21,22,38]. The coexistence of stellate, bipolar, branched, chain-like, and dense network-forming patterns demonstrates morphological variability within the enriched culture under the applied in vitro conditions but does not establish stable or functionally distinct telocyte subpopulations. Comparable morphological variability has been reported in descriptive studies of testicular telocytes in rodents and other mammals [10,11,12,13,18,23] and in studies demonstrating morphological plasticity of telocytes in vitro [33,34,38]. These comparisons provide relevant context but do not demonstrate equivalence between the observed culture-associated patterns and distinct in vivo cellular states. Future studies combining phenotypic, ultrastructural, quantitative, molecular, and functional approaches will be required to determine whether the observed culture-associated morphological patterns have biological and functional relevance [19,24]. This approach provides a methodological foundation for future studies on telocyte biology in the male gonad.

## 5. Conclusions

This study presents a flow cytometry-assisted strategy for enriching a rat testicular stromal cell population displaying immunophenotypic and morphological features consistent with telocytes. The introduction of an empirical αSMA-associated exclusion step was intended to reduce PTMC-like contamination and facilitate morphological evaluation of the enriched cells. Several recurring morphological patterns were observed under in vitro conditions; however, these patterns cannot be assumed to represent stable in vivo or functionally distinct telocyte subpopulations.

The absence of expanded post-sorting immunophenotyping, including assessment of PDGFRα and vimentin, as well as transmission electron microscopy, quantitative morphometry, and functional assays, limits the completeness of cell characterization and the interpretation of the biological significance of the observed heterogeneity. The proposed approach therefore provides a methodological basis for future optimization and for phenotypic, ultrastructural, quantitative, and functional studies of rat testicular telocytes. Following further phenotypic and ultrastructural validation, this culture model may provide an experimental platform for investigating telocyte interactions with other testicular cell populations and their potential contribution to the organization and regulation of the male gonadal microenvironment.

## Data Availability

The data presented in this study are contained within the article. Raw microscopy images and flow cytometry data are available upon reasonable request from the corresponding author.
